# Dynamin-related protein 1 has membrane constricting and severing abilities sufficient for mitochondrial and peroxisomal fission

**DOI:** 10.1038/s41467-018-07543-w

**Published:** 2018-12-07

**Authors:** Sukrut C. Kamerkar, Felix Kraus, Alice J. Sharpe, Thomas J. Pucadyil, Michael T. Ryan

**Affiliations:** 1 Indian Institute of Science Education and Research, Dr. Homi Bhabha Road, Pashan, Pune, 411008 Maharashtra India; 2 Department of Biochemistry and Molecular Biology, Monash Biomedicine Discovery Institute, Monash University, 3800 Melbourne, Australia

## Abstract

Dynamin-related protein 1 (Drp1) is essential for mitochondrial and peroxisomal fission. Recent studies propose that Drp1 does not sever but rather constricts mitochondrial membranes allowing dynamin 2 (Dnm2) to execute final scission. Here, we report that unlike Drp1, Dnm2 is dispensable for peroxisomal and mitochondrial fission, as these events occurred in Dnm2 knockout cells. Fission events were also observed in mouse embryonic fibroblasts lacking Dnm1, 2 and 3. Using reconstitution experiments on preformed membrane tubes, we show that Drp1 alone both constricts and severs membrane tubes. Scission required the membrane binding, self-assembling and GTPase activities of Drp1 and occurred on tubes up to 250 nm in radius. In contrast, Dnm2 exhibited severely restricted fission capacity with occasional severing of tubes below 50 nm in radius. We conclude that Drp1 has both membrane constricting and severing abilities and is the dominant dynamin performing mitochondrial and peroxisomal fission.

## Abstract (web summary)

Drp1 and Dnm2 have been implicated in mitochondrial fission events, although their specific activities in constriction and scission have been unclear. Here, the authors demonstrate that Drp1 is sufficient to constrict and sever mitochondrial and peroxisomal membranes in the absence of Dnm proteins.

## Introduction

Mitochondria are double-membrane organelles and their division is linked to organelle quality control, segregation of mtDNA, mitophagy, and apoptosis^1^. Mitochondrial division is regulated by a plethora of cellular factors including the metabolic state of the cell, reactive oxygen species, calcium signaling, and ER constriction^2,3^. Peroxisomes, while sharing a dual origin from both the ER and mitochondria^4^, are single-membrane organelles that catalyze the breakdown of very long chain fatty acids through beta-oxidation^5^. However, cellular cues that regulate their division are relatively less clear. Both mitochondrial and peroxisomal fission is facilitated by dynamin-related protein 1 (Drp1)^6–10^, a member of the dynamin superfamily of proteins that self-assemble as helical scaffolds and utilizes the energy from GTP hydrolysis to constrict and remodel tubular membrane intermediates^11,12^. Drp1 shares several architectural principles with the classical dynamins involved in vesicle budding, including the GTPase domain, the bundle signaling element, and the stalk region^13^. However, in contrast to the classical dynamins, Drp1 lacks the specialized pleckstrin-homology domain required for membrane binding and insertion^14,15^. Instead, Drp1 contains the B-insert, a variable and unstructured 100 residue-long loop at the end of the stalk, that binds adapter proteins and the mitochondrial lipid cardiolipin^16,17^. Interestingly, evolutionary analysis suggests that present-day lineages of dynamin could have evolved from an ancestral bifunctional dynamin capable of mitochondrial and vesicle scission^18^.

While adapter proteins such as mitochondrial fission factor (Mff), mitochondrial dynamics protein 49 (MiD49) and MiD51 function to recruit Drp1 from the cytoplasm^17,19^, mitochondrial fission appears to follow pre-constriction of the organelle by the ER^20^. In addition, a recent study puts the classical dynamin 2 (Dnm2) at the center of mitochondrial fission since knockdown of Dnm2 resulted in significant mitochondrial elongation, with Drp1 accumulating at both ends of highly constricted tubular membrane intermediates^21^. This pointed toward the possibility that Dnm2 severs the final single-membrane tube between two Drp1 scaffolds and was supported by Dnm2 localization studies. This finding is consistent with all in vitro reconstitution studies carried out so far, which indicate that the most Drp1 is capable of is to self-assemble on membranes to stabilize constricted tubular intermediates but not execute final membrane scission^13,22–26^.

Here we use a variety of experimental approaches to investigate membrane fission capabilities of Dnm2 and Drp1 in peroxisomal and mitochondrial fission. We show that Drp1 is critical for both mitochondrial and peroxisomal fission, while other cytosolic dynamins are dispensable. In addition, reconstitution experiments show that Drp1 can bind, constrict, and sever preformed membrane tubules alone in vitro.

## Results

### Drp1 can drive peroxisomal fission in the absence of Dnm2

**Fig. 1:**
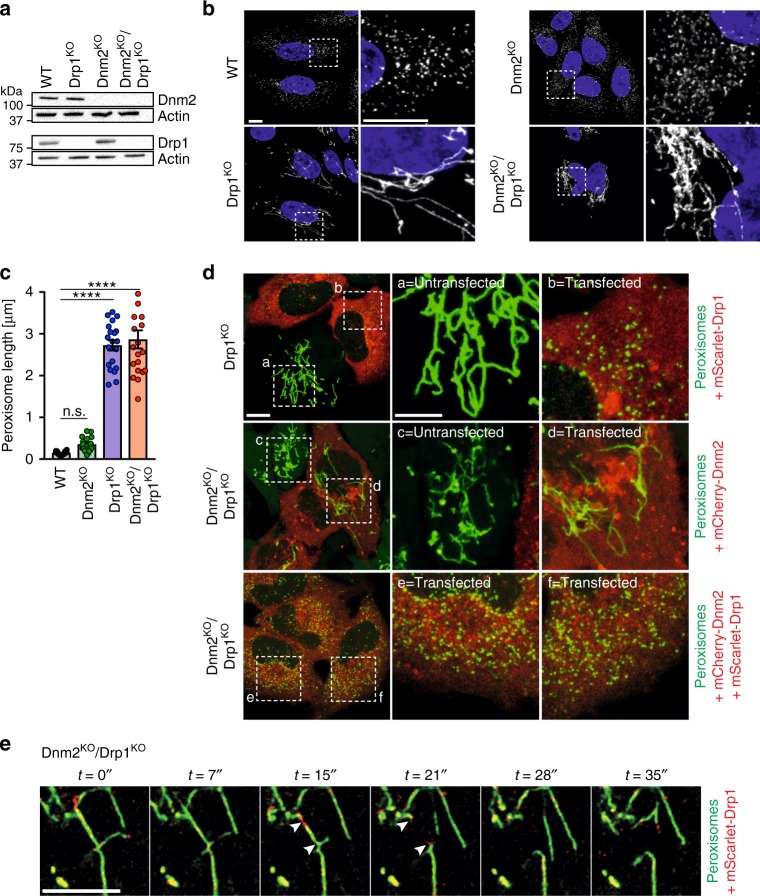
Loss of Dnm2 does not affect division of peroxisomes. **a** Western blot analysis of HeLa WT, Dnm2^KO^, Drp1^KO^, and Dnm2^KO^/Drp1^KO^ whole cell lysates. Actin was used as a loading control. **b** Confocal images of HeLa WT, Drp1^KO^, Dnm2^KO^, and Dnm2^KO^/Drp1^KO^ cells subjected to immunofluorescence and stained for peroxisomes (Pex14, white). Blue marks the nucleus. An enlargement of the hatched box is shown on the right of each panel. Scale bar = 10 µm. **c** Computational quantification of average peroxisome length in cell lines. *n*(WT) = 20 cells, *n*(Dnm2^KO^) = 20 cells, *n*(Drp1^KO^) = 20 cells, *n*(Dnm2^KO^/Drp1^KO^) = 18 cells. Data obtained from three independent experiments. Data represents the mean ± S.E.M.; n.s., not significant; ****p < 0.0001. One-way ANOVA with multiple comparisons. **d** Confocal live-cell image stills of HeLa Drp1^KO^ and Dnm2^KO^/Drp1^KO^ stably expressing Dendra2-SKL (green) and transfected with mCherry-Dnm2 (red) and/or doxycycline-inducible mScarlet-Drp1 (red) constructs. Enlargements of the hatched boxes depicting peroxisomal morphology in untransfected and transfected cells are shown on the right. Scale Bar = 10 µm and 5 µm (magnifications). **e** Confocal live-cell image stills of peroxisome fission events in HeLa Dnm2^KO^/Drp1^KO^ cells stably expressing Dendra2-SKL and doxycycline-inducible mScarlet-Drp1. Scale bar = 5 µm

Given the model proposing the cooperation between Drp1 and Dnm2 in mitochondrial division, we sought to address if the same was applicable for peroxisomes. To study this, we created CRISPR/Cas9-mediated knockouts of Dnm2, Drp1, and a Dnm2/Drp1 double knockout in HeLa cells. Sequencing of alleles confirmed gene disruption in the clonal knockout (KO) cell lines (Supplementary Table S1) and was supported by western blot analysis (Fig. 1a). Dnm2^KO^ and Dnm2^KO^/Drp1^KO^ cells showed defects in endocytosis, as previously established (Supplementary Figure 1A)^27^. Interestingly, some reduction in endocytosis was also seen upon loss of Drp1 (Supplementary Figure 1A), consistent with results from a previous study^28^, although this may be isoform-specific^29^. To assess peroxisomal morphology, we performed immunofluorescence labeling of the organelle (Fig. 1b). Loss of Drp1 caused peroxisomes to form long thread-like structures, characteristic of fission defects, as previously reported^10^. In contrast, peroxisomes remained short in Dnm2^KO^ cells and were similar in length to those seen in control cells (Fig. 1b, c). Interestingly, loss of Dnm2 led to an increase in the total numbers of peroxisomes (Supplementary Figure 1B), which may indicate increased biogenesis and/or decreased turnover of these organelles. Loss of both Drp1 and Dnm2 was not cumulative; Dnm2^KO^/Drp1^KO^ cells displayed no additional changes in peroxisome length in comparison to cells lacking Drp1 alone (Fig. 1b, c). Transient re-expression of Drp1 (fused to red fluorescent protein, mScarlet) into Drp1^KO^ cells led to the restoration of WT-like, short peroxisomes (Fig. 1d). In contrast, expression of Dnm2 (fused to mCherry) in Dnm2^KO^/Drp1^KO^ cells was unable to rescue peroxisomal fission defects; only after the additional re-expression of Drp1 were peroxisomes converted to their spherical shape (Fig. 1d). Finally, time-lapse imaging of newly expressed mScarlet-Drp1 in Dnm2^KO^/Drp1^KO^ cells revealed the presence of Drp1 foci at sites of peroxisomal fission (Fig. 1e, Supplementary Movie 1). These results indicate that Drp1 can function independently of Dnm2 in executing peroxisomal fission.

### Drp1 but not Dnm2 is essential for mitochondrial fission

**Fig. 2:**
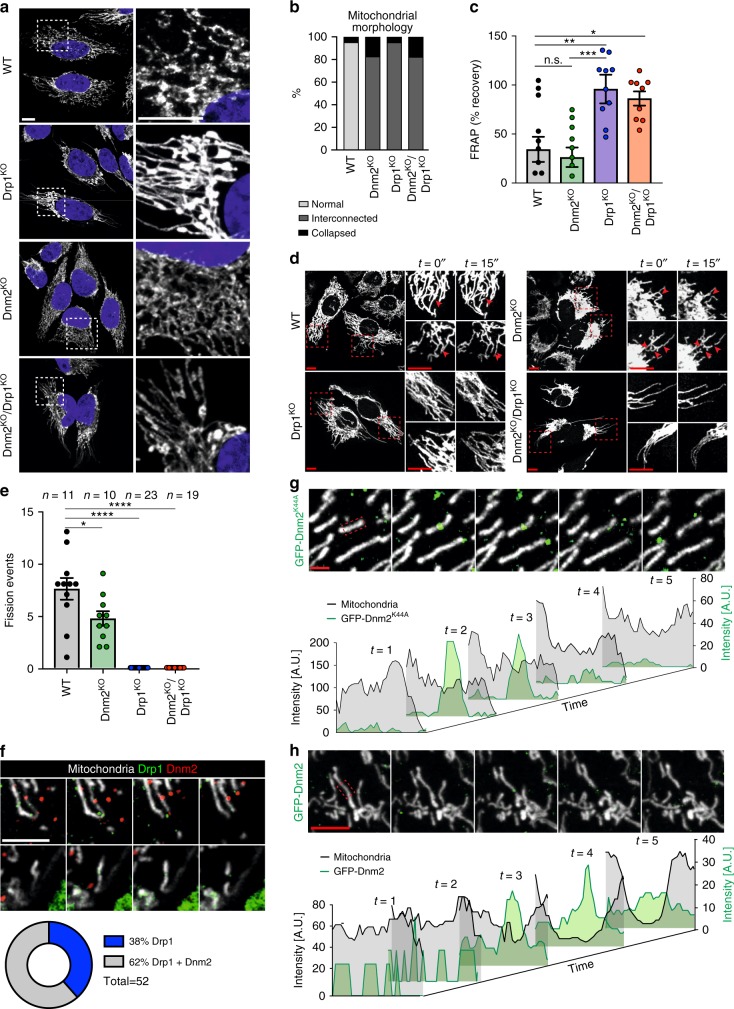
Drp1 is dominant in mitochondrial fission. **a** Confocal images of HeLa WT, Drp1^KO^, Dnm2^KO^, and Dnm2^KO^/Drp1^KO^ cells subjected to immunofluorescence and stained for cytochrome c (white). Blue marks the nucleus. An enlargement of the hatched box is shown on the right of each panel. Scale bar = 10 µm. **b** Qualitative scoring of mitochondrial morphology. *n*(WT) = 41 cells; *n*(Dnm2^KO^) = 69 cells; *n*(Drp1^KO^) = 142 cells; *n*(Dnm2^KO^/Drp1^KO^) = 73 cells. **c** FRAP analysis of mtDendra2 in WT, Drp1^KO^, Dnm2^KO^, and Dnm2^KO^/Drp1^KO^ HeLa cells. *n*(WT) = 12 cells; *n*(Dnm2^KO^) = 11 cells; *n*(Drp1^KO^) = 12 cells; *n*(Dnm2^KO^/Drp1^KO^) = 10 cells. Data obtained from three independent experiments. Data represents the mean ± S.E.M.; n.s., not significant; *p < 0.05, **p < 0.005, ***p < 0.001. One-way ANOVA with multiple comparisons. **d** Representative confocal image frames depicting fission events for each cell line from (**e**). An enlargement of the hatched box is shown on the right of each panel. Scale bar = 10 µm. *n*(WT) = 11 cells; *n*(Dnm2^KO^) = 10 cells; *n*(Drp1^KO^) = 23 cells; *n*(Dnm2^KO^/Drp1^KO^) = 19 cells. **e** Number of individual mitochondrial fission events in HeLa WT, Drp1^KO^, Dnm2^KO^, and Dnm2^KO^/Drp1^KO^ cells counted over a 7.5 min period. *n*(WT) = 11 cells; *n*(Dnm2KO) = 10 cells; *n*(Drp1KO) = 24 cells; *n*(Dnm2KO/Drp1KO) = 20 cells. Data obtained from three independent experiments. Data represents the mean ± S.E.M.; *p < 0.01, ****p < 0.0001. One-way ANOVA with multiple comparisons. **f** Mitochondrial fission events in HeLa Dnm2^KO^ cells expressing mCherry-Dnm2 and GFP-Drp1. Mitochondrial fission events showing both Drp1 and Dnm2 (top panel) and Drp1 alone (bottom panel) at the fission site. Scale bar = 5 µm. Analysis of mitochondrial fission events (*n* = 14 cells; 52 fission events). **g** Live-cell confocal stills of HeLa cells expressing GFP-Dnm2^K44A^ and stained with MitoTracker Red. Intensities of a mitochondrial tubule and GFP-Dnm2^K44A^ foci (red hash box) over times are plotted below. Scale bar = 5 µm. **h** As for panel **g** but for GFP-Dnm2 where a fission event is observed

Since Dnm2 has been suggested to be a key player in mitochondrial division^21^, we also assessed mitochondrial morphology in the KO cell lines. As previously reported^8^, loss of Drp1 showed clear interconnected, hyperfused mitochondrial tubules, often displaying large and bulbous mitochondria in the vicinity of the nucleus (Fig. 2a). In contrast, cells lacking Dnm2 showed a somewhat different phenotype, with apparent dense mitochondrial networks and lacking the bulbous mitochondria (Fig. 2a, b and Supplementary Figure 1C). Due to the dense nature of these networks, we were unable to use morphometric approaches to reliably determine differences in mitochondrial dynamics. We therefore created stable cell lines expressing mitochondria-targeted, photoconvertible Dendra2 (mtDendra2) and used them for FRAP-like experiments as a measure of mitochondrial interconnectivity (Supplementary Movies 2–5). In Drp1^KO^ and Dnm2^KO^/Drp1^KO^ cell lines, recovery of green mtDendra2 was observed 5 min after FRAP irradiation, in contrast to WT and Dnm2^KO^ cells (Fig. 2c). This data implies that in Drp1^KO^ and Dnm2^KO^/Drp1^KO^ cells, mitochondria are more fused (due to fission defects) than in WT and Dnm2^KO^ cells. In addition, mitochondrial fission events still occurred in Dnm2^KO^ cells (over a 7.5 min time frame), but the number was slightly reduced compared to WT cells (Fig. 2d, e). Cells lacking Drp1 had no detectable fission events (Fig. 2d, e). We conclude that while loss of Dnm2 leads to alterations in the mitochondrial network, fission events still occur and are dependent on Drp1.

The reduction in mitochondrial fission events in Dnm2^KO^ cells, especially since no significant compensation in Drp1 levels were found after loss of Dnm2 (Supplementary Figure 1E), suggests a role for Dnm2 in mitochondrial fission. Indeed, of the 52 fission events analyzed in Dnm2^KO^ cells, 32 showed the coincident presence of mCherry-Dnm2 and GFP-Drp1 while 20 showed the absence of mCherry-Dnm2 but presence of GFP-Drp1 (Fig. 2f). However, further analysis with a dominant-negative GFP-tagged Dnm2^K44A^ mutant^30^ revealed that the requirement for Dnm2 may be bypassed during fission. HeLa and HEK293T cells expressing GFP-tagged Dnm2^K44A^ displayed mitochondrial and peroxisomal morphologies that were largely similar to control cells (Supplementary Figure 1F, G). Consistent with Dnm2’s role in clathrin-mediated endocytosis, expression of GFP-tagged Dnm2^K44A^ showed impaired transferrin uptake, comparable to that seen in Dnm2^KO^ cell lines (Supplementary Figure 1D)^31^. Interestingly, cells expressing this mutant displayed a somewhat denser mitochondrial network, resembling the phenotype of Dnm2^KO^ cell lines. However, live-cell microscopy revealed that both WT Dnm2 and the Dnm2^K44A^ mutant localized to mitochondria, but only Dnm2^K44A^ bound transiently to mitochondria without remodeling of the network (Fig. 2g, h), as is seen following expression of Drp1 dominant-negative mutants^32^. The Dnm2^K44A^ mutant is defective in GTP hydrolysis and should remain locked in the constricted state on membranes^33^. Thus if Dnm2 were critical for mitochondrial fission, GFP-Dnm2^K44A^ foci should have remained static on a constricted prefission intermediate on mitochondria reflecting a stalled fission pathway, which is not the case.

Finally, we tested whether there was a different requirement for Dnm2 and Drp1 in cells undergoing global fission due to dissipation of mitochondrial membrane potential^34^. HeLa cells were treated with the uncoupler FCCP, fixed and immunofluorescently labeled for mitochondria and peroxisomes (Supplementary Figure 2A). In WT cells, mitochondrial fragmentation was observed, while cells lacking Drp1 showed swollen but not fragmented mitochondria, consistent with previous studies^8^. In Dnm2^KO^ cells, mitochondria were fragmented similarly to WT cells. As expected, peroxisomal morphology was not affected by FCCP addition (Supplementary Figure 2A). The alterations in the mitochondrial network and peroxisome numbers upon loss of Dnm2 also suggest that this protein is involved in organelle maintenance. Rescue of the Dnm2^KO^ cells with a GFP-tagged version of Dnm2 restored the dense mitochondrial network to wild-type morphology (Supplementary Figure 2B). Given the relevance of ER-mitochondria contact sites for mitochondrial fission^20^, we assessed ER morphology using BFP-Sec61^35^ and found no apparent differences in such contacts across control, Dnm2^KO^, and Drp1^KO^ cells (Supplementary Figure 2C). However, the ER network in both Drp1^KO^ and Dnm2^KO^ cells appeared less compartmentalized and more diffuse in comparison to WT cells (Supplementary Figure 2C). The reasons for this is not clear, but cellular and cytoskeletal responses to the loss of Drp1 or Dnm2 are feasible explanations that could be pursued in the future.

### Organellar fission occurs in cells lacking dynamins 1,2,3

**Fig. 3:**
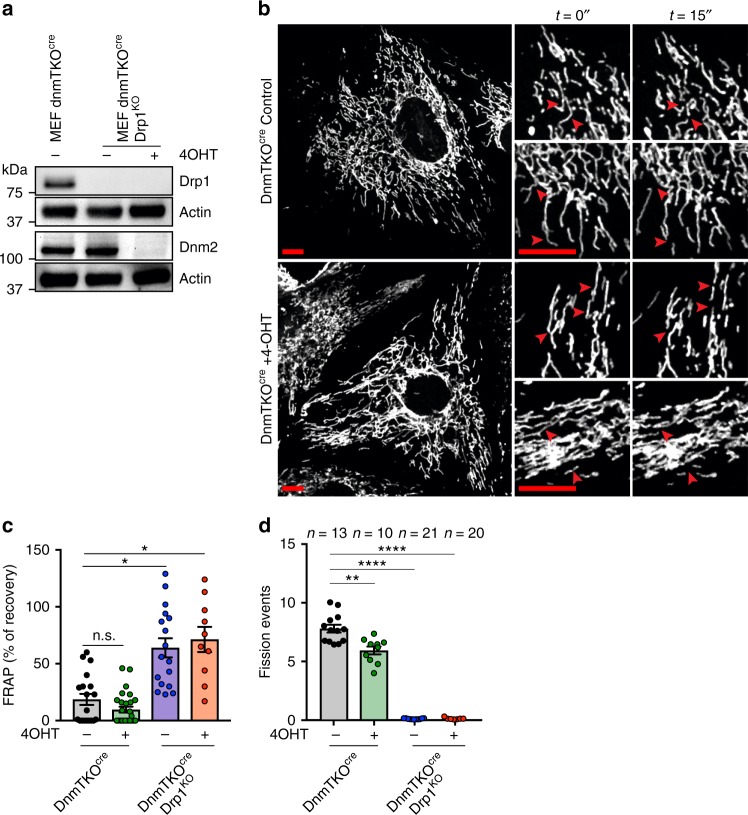
Loss of Drp1 is dominant to loss of dynamins 1,2,3. **a** Western blot analysis of MEF DnmTKO^cre^, DnmTKO^cre^+Drp1^KO^ control and 4-OHT induced cells on whole cell lysates. Actin was used as a loading control. **b** Representative confocal image frames depicting mitochondrial morphology and fission events of MEF DnmTKO^cre^ control and knockout (4-OHT induced) cells. Two enlargements on the right of each panel show fission events over the course of 15 s (red arrowheads). Scale bar = 10 µm. **c** FRAP analysis of mtDendra2 in MEF DnmTKO^cre^, DnmTKO^cre^+Drp1^KO^ control and 4-OHT induced cells. *n*(DnmTKO^cre^) = 23 cells; *n*(DnmTKO^cre^ + 4OHT) = 31 cells; *n*(DnmTKO^cre^ + Drp1^KO^) = 26 cells; *n*(DnmTKO^cre^+Drp1^KO^+4OHT) = 23 cells. Data obtained from three independent experiments. Data represents the mean ± S.E.M.; n.s., not significant; *p < 0.05. One-way ANOVA with multiple comparisons. **d** Number of mitochondrial fission events in MEF DnmTKO^cre^, DnmTKO^cre^+Drp1^KO^ control and 4-OHT induced cells counted over a 7.5 min period. *n*(DnmTKO^cre^) = 13 cells; *n*(DnmTKO^cre^+4OHT) = 10 cells; *n*(DnmTKO^cre^ + Drp1KO) = 21 cells; *n*(DnmTKO^cre^ + Drp1KO + 4OHT) = 20 cells. Data obtained from three independent experiments. Data represents the mean ± S.E.M.; **p < 0.01, ****p < 0.0001. One-way ANOVA with multiple comparisons

Compared to the strong mitochondrial and peroxisomal fission defects in Drp1^KO^ cell lines, the lack of a clear dependency for Dnm2 may be due to overlapping functions of dynamins 1 and/or 3. Indeed, while Dnm1 and Dnm3 are enriched in neuronal tissues^36,37^, western blot analysis showed that Dnm1 is expressed in HeLa cells (Supplementary Figure 3A). We therefore analyzed the previously established 4-OHT cre-inducible dynamin 1,2,3 triple knockout MEF cell line (now called DnmTKO^cre^)^27^. Following induction of cre-expression with 4-OHT, mitochondrial and peroxisomal networks appeared similar to that seen in control cells (Fig. 3b, Supplementary Figure 3B). Additional knockout of Drp1 in these cells resulted in the formation of hyperfused mitochondria indicating that the fusion machinery is still operative in these cells (Fig. 3a, Supplementary Figure 3C). To determine the potential differences in mitochondrial dynamics after loss of dynamins 1,2,3, we expressed mtDendra2 and performed FRAP and mitochondrial fission counting experiments. FRAP experiments revealed a slight reduction in mtDendra2 recovery between control and dynamin 1,2,3 KO cells, while the additional loss of Drp1 showed a significant increase in recovery, consistent with a hyperfused mitochondrial phenotype (Fig. 3c). Movement of photoactivated mtDendra to the opposite side of the mitochondrial network was also accelerated by the loss of Drp1 while this was not the case following loss of dynamins 1,2,3 (Supplementary Figure 3D). Nevertheless, like HeLa Dnm2^KO^ cells, loss of dynamins 1,2,3 resulted in an overall decrease in mitochondrial fission events (Fig. 3d). Finally, stress-induced mitochondrial fission using FCCP was investigated under live-cell conditions. In both control and DnmTKO^cre^ cells, mitochondrial fission events were still observed in a 7-min time-window (fission events highlighted with red arrows) (Supplementary Figure 3E). Together therefore, our results are consistent with Drp1 being the dominant dynamin member for both mitochondrial and peroxisomal fission.

### Tubular membrane templates to analyze Drp1 functions

**Fig. 4:**
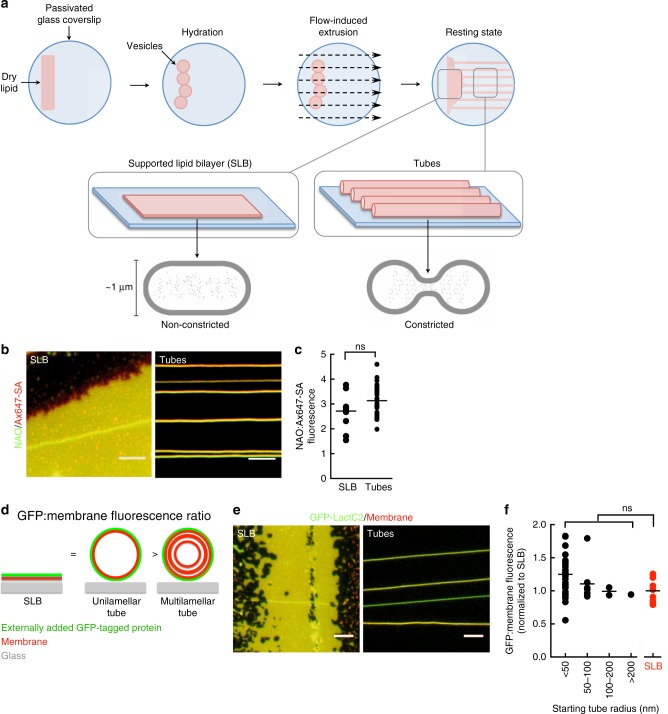
Template characteristics. **a** Schematic of the membrane template. **b** Representative images of NAO and Alexa647-labeled streptavidin (SA) fluorescence on the SLB and tubes composed of CL:DOPC (25:75 mol%). Scale bar = 10 µm. **c** Ratios of NAO and Alexa647-labeled streptavidin (SA) fluorescence on the SLB and tubes composed of CL:DOPC (25:75 mol%); ns, not significant, Mann–Whitney test. *n*(SLB) = 19 and *n*(tube) = 25 samplings from a single template preparation. **d** Schematic showing the expected fluorescence ratios of an externally added GFP-tagged protein that binds the membrane on the SLB, unilamellar and multilamellar tubes. **e** Representative images showing GFP-LactC2 distribution on the SLB and tubes. Scale bar = 10 µm. **f** Ratios of GFP-LactC2 and membrane fluorescence on tubes of varying sizes compared to that seen on the SLB; ns, not significant, Mann–Whitney test. *n*(<50 nm) = 33, *n*(50–100 nm) = 8, *n*(100–200 nm) = 2, *n*(>200 nm) = 1, and *n*(SLB) = 10 samplings from a single template preparation

Previous in vitro work using giant unilamellar vesicles or liposomes has demonstrated a role for Drp1 in membrane scaffolding and tubulation^13,23,25,26,38^. To directly address Drp1 and Dnm2 functions in membrane fission, we focused our analysis on preformed lipid tubules that better mimic the tubular topology of mitochondria and peroxisomes. We used purified recombinant human Drp1 (Supplementary Figure 4A), which we first confirmed was functionally active by assaying its stimulated basal GTPase activity in the presence of liposomes composed of 25 mol% cardiolipin (CL) in a dioleolyphosphatidyl choline (DOPC) background^38^ (Supplementary Figure 4B). We next prepared a membrane template of identical lipid composition that displays an array of membrane tubes connected to a planar supported lipid bilayer (SLB) (Fig. 4a)^39^. Templates contained trace amounts (1 mol%) of a membrane curvature-insensitive fluorescent lipid *p*-Texas Red DHPE to visualize the membrane and estimate tube sizes. Tube radii are estimated using a fluorescence-based methodology using the SLB as an in situ calibration standard (Supplementary Figure 5). Depending on the initial buffer flow used to extrude the membrane reservoir, tubes in such preparations have radii in the 10–400 nm range, which mimic the range of mitochondrial topologies (intact and constricted) observed in cells^20^. Control experiments monitoring the distribution of an anionic lipid sensor nonyl acridine orange (NAO) and a membrane curvature-insensitive biotinylated lipid labeled with Alexa647-streptavidin^40^ revealed CL to be evenly distributed between the tubes and SLB (Fig. 4b, c). To test membrane lamellarity, we assayed binding of an externally added fluorescent protein to a specific lipid incorporated in the starting lipid mix used to form the templates. Binding or accessibility was measured by estimating the ratio of protein to membrane fluorescence. Since the protein is expected to bind lipids only displayed on the outermost lamellae, unilamellar tubes would show a ratio similar to that seen for the SLB while multilamellar tubes would show a ratio lower than seen for the SLB (Fig. 4d). Since a CL-binding protein or domain was not available, we used templates composed of 15 mol% PS and the phosphatidylserine-specific LactC2 domain tagged with GFP (GFP-LactC2)^41^ (Fig. 4e). These experiments revealed no significant difference between the GFP to membrane fluorescence ratio for tubes of different sizes and the SLB, which indicates that the tubes in the templates are unilamellar (Fig. 4f).

To analyze Drp1 dynamics on these templates, we first tested various fluorescent constructs in GTPase assays using CL-containing liposomes. Surprisingly, the N-terminal GFP-tagged Drp1 construct, which localizes to peroxisomes and mitochondria (Figs. 1e, 2h) and rescues the hyperfused mitochondrial phenotype seen in Drp1^KO^ cell lines (Fig. 1d), showed a high basal GTPase activity that was not stimulated with CL-containing liposomes (Supplementary Figure 4B). These results imply that cellular factors (such as the presence of mitochondrial adapter proteins) could have rescued defects evident in the more stringent in vitro assays with this construct. Drp1 labeled extrinsically with a thiol-reactive Alexa fluorophore, as reported earlier^22,23,38^, showed complete loss of assembly-stimulated GTPase activity (Supplementary Figure 4B). In contrast, Drp1 tagged with GFP at the C-terminus (Drp1-GFP) showed assembly-stimulated GTPase activity but to an extent significantly lower than seen with WT (Supplementary Figure 4B). Since this was the only construct that showed assembly-stimulated GTPase activity like WT on CL-containing liposomes, we used it for further experiments but only in an equimolar mixture with WT Drp1, hereafter referred to as Drp1 ± GFP.

### Membrane binding and self-assembly of Drp1

**Fig. 5:**
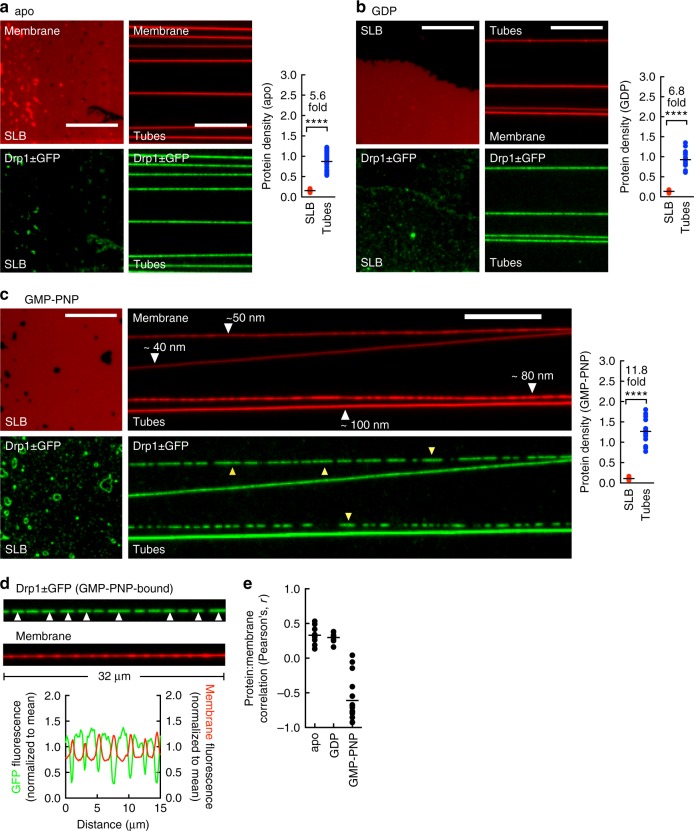
Self-assembly of Drp1. **a** Representative images of Drp1 ± GFP on the SLB and tubes in the apo (**a**), GDP-bound (**b**), and GMP-PNP-bound (**c**) states. Scale bar = 10 µm. Also shown are ratios of Drp1 ± GFP to membrane fluorescence on the SLB and tubes; ****p < 0.0001, Mann–Whitney test. *n*(SLB) = 28 and *n*(tube) = 25 from a single experiment for apo. *n*(SLB) = 21 and *n*(tube) = 16 from a single experiment for GDP. *n*(SLB) = 21 and *n*(tube) = 15 from a single experiment for GMP-PNP. Yellow arrowheads mark clusters of Drp1 ± GFP on tubes in the GMP-PNP-bound state. **d** Representative images and associated line profiles showing distribution of Drp1 ± GFP with GMP-PNP. White arrowheads mark clusters of Drp1 ± GFP. **e** Pearson’s coefficient of correlation between Drp1 ± GFP and membrane fluorescence. *n*(apo) = 10, *n*(GDP) = 10, and *n*(GMP-PNP) = 16 tubes from a single experiment

Templates were incubated with Drp1 ± GFP alone or with a non-hydrolyzable GTP analog GMP-PNP or with GDP for 10 min and excess unbound protein was washed out. Remarkably, under all these conditions, we found the protein to be localized selectively on the tubes compared to the planar SLB (Fig. 5a–c), implying that Drp1 displays an intrinsic preference for binding membranes of high curvature. Of note, when these experiments were carried out with the fluorescent constructs added without WT, only Drp1-GFP showed binding to membranes thus explaining why it was the only fluorescent construct that showed membrane-stimulated GTPase activity (Supplementary Figure 4C). Together, these results validate its use as a fluorescent reporter for in vitro assays. On tubes, Drp1 ± GFP was uniformly distributed in the apo and GDP-bound states (Fig. 5a, b) but occasionally formed clusters with GMP-PNP (Fig. 5c, yellow arrowheads). Clustering did not depend on the tube size since often in the same microscope field both thin and thick tubes showed a uniform distribution while tubes of intermediate sizes showed clusters (Fig. 5c, yellow arrowheads). We are currently unsure as to what causes this variability in distribution. When looked at against adjacent regions on the same tube devoid of any protein, the clusters appeared to reduce fluorescence of the underlying tube (Fig. 5d, white arrowheads and line profiles). Together, this is apparent in a Pearson’s correlation analysis of Drp1 ± GFP and membrane fluorescence where the apo and GDP-bound states showed little correlation while the GMP-PNP-bound state showed a range of values signifying little to significantly negative correlation (Fig. 5e). Since the membrane tube is diffraction-limited, reduced tube fluorescence signifies constriction and is consistent with differences seen in the cryoEM reconstructions of the protein in the apo- and GMP-PNP-bound states^24,42^. Since the uniform distribution on some tubes with GMP-PNP could reflect a single long cluster of the protein, together we interpret these results to indicate that GTP-binding induces Drp1 to form membrane-active scaffolds that constrict the tube to variable extents. This is in contrast to the classical dynamins that constrict the underlying membrane tube in the apo state and to a defined extent^43–45^.

### GTP hydrolysis-dependent membrane fission by Drp1

**Fig. 6:**
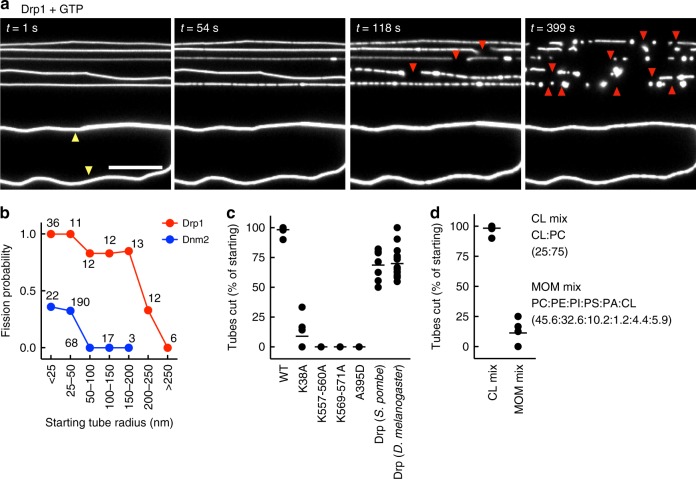
Drp1- and Dnm2-catalyzed membrane fission. **a** Frames from a time-lapse movie showing Drp1-catalyzed fission. Red arrowheads mark site of fission, yellow arrowheads mark tubes that remain uncut. Scale bar = 10 µm. **b** Fission probability with Drp1 and Dnm2 as a function of starting tube radius. *n*(tubes) for each size range is indicated in the plot. **c** Analysis of Drp1-catalyzed fission with the indicated mutants and orthologs. *n*(WT) = 10, *n*(K38A) = 11, *n*(K557–560A) = 5, *n*(K569–571A) = 3, *n*(A395D) = 6, *n*(Drp *S. pombe*) = 7, and *n*(Drp *D. melanogaster*) = 16 microscope fields of a single experiment where each field displayed 30–35 tubes. **d** Analysis of Drp1-catalyzed fission on templates of the indicated lipid composition. *n*(CL mix) = 8 and *n*(MOM mix) = 7 microscope fields from a single experiment where each field displayed 30–35 tubes

Recent cryoEM analysis of Drp1 in solution reveals two self-assembled states; one as a helical scaffold with GMP-PNP and the other as rings with GTP^42^. In addition, recent EM analysis of co-filaments of Drp1 and the adapter protein MiD49 reveal that GTP hydrolysis causes dramatic structural alterations that result in filament shortening and curling of Drp1 oligomers into constricted and closed rings^46^. To analyze if such structural alterations can affect membrane remodeling, we flowed in Drp1 with GTP to the templates. Remarkably, this caused a dramatic severing of membrane tubes (Fig. 6a, Supplementary Movie 6). Tubes in solution also underwent severing (see Supplementary Movie 7), implying that their surface-attachment has little influence on Drp1’s fission capacity. However, not all tubes in these templates were cut (e.g., tubes marked by yellow arrowheads in Fig. 6a). A systematic analysis revealed fission to be sensitive to the starting tube size; tubes up to 200 nm radius showed a high fission probability while those between 200–250 nm radius showed lower fission probability and those above 250 nm radius were refractory to fission (Fig. 6b). These results are the first to reveal an intrinsic capacity of Drp1 to constrict and sever tubes and also inform us of the role of membrane topology in regulating the fission reaction. In light of the recently proposed models suggesting a collaboration between the classical and mitochondrial dynamins in mitochondrial division^21^, we independently analyzed Dnm2’s ability to sever such templates. Remarkably, we find Dnm2 to be severely restricted in fission; only tubes below 50 nm radius showed severing and that too with a low probability while tubes above this size limit were completely refractory to fission (Fig. 6b).

To further analyze determinants for the Drp1-catalyzed fission process, we turned to templates with tubes ranging from 10 to 50 nm in radius that showed robust fission by Drp1 (Fig. 6b). We found that the GTPase-defective K38A mutant^6^, B-insert mutants defective in membrane binding (K557A; K506A and K569A; K571A)^16^ and the self-assembly defective A395D mutant^47^ either showed severely compromised or complete absence of fission indicating that fission requires Drp1’s membrane binding, self-assembly, and stimulated GTP hydrolysis activities (Fig. 6c). Importantly, the fission activity appears to be evolutionarily conserved since both the yeast and fly orthologs of Drp1 displayed robust fission of membrane tubes in presence of GTP (Fig. 6c). Under these simplified reconstituted conditions, fission required an excess of CL, possibly to facilitate Drp1 binding to the membrane, since tubes formed of a complex lipid mix that mimics the mitochondrial outer membrane were refractory to fission^48^ (Fig. 6d).

**Fig. 7:**
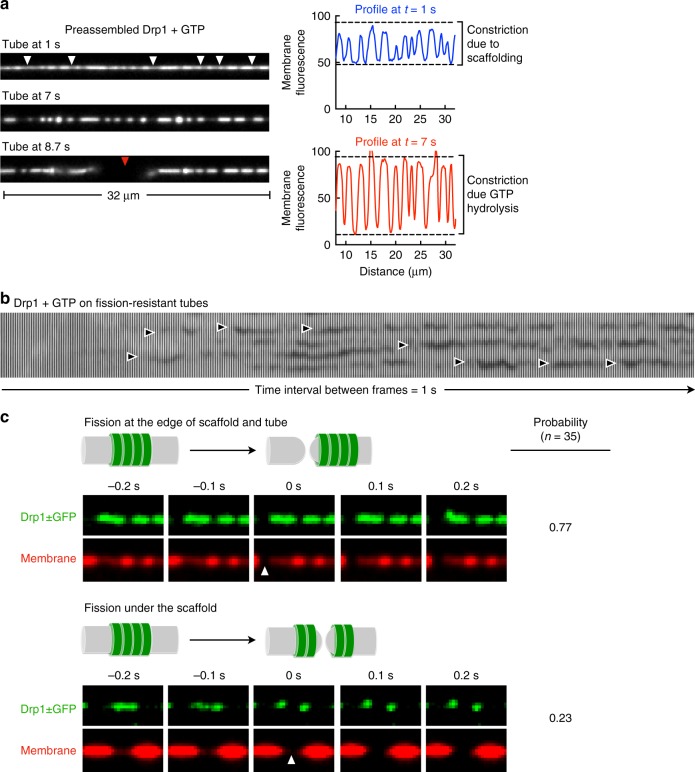
Mechanism of Drp1-catalyzed fission. **a** Frames from a representative time-lapse movie showing the effect of GTP addition to preassembled Drp1 scaffolds. Also shown are line profiles of tube fluorescence at the indicated time points. White arrowheads mark constrictions formed by Drp1 scaffolds, red arrowhead marks the site of fission. **b** Montage of tube images from a representative experiment shown decline and recovery of tube fluorescence at sites marked by black arrowheads. **c** Frames from a time-lapse movie showing the distribution of Drp1 ± GFP (green) on the tube (red) under conditions where the scaffold remains intact (top) and when it undergoes splitting (bottom) after fission. White arrowhead marks the site of tube fission. Also shown is the probability of these two types of fission events

To analyze the fission mechanism, we first flowed in Drp1 with GMP-PNP and allowed it to forms scaffolds (referred to as preassembled Drp1, Fig. 7a, white arrowheads, tube at *t* = 1 s, blue profile) and then flowed in GTP. We focused on tubes that showed clusters of Drp1 since we could assess the effect of GTP hydrolysis on the tube under the scaffold and compare it to that adjacent to the scaffold. Remarkably, GTP arrival led to the tube undergoing further constriction (Fig. 7a, tube at *t* = 7 s, red profile, see Supplementary Movie 8), before fission took place (Fig. 7a, red arrowhead). On wide tubes, which resisted severing, addition of Drp1 with GTP led to cycles of decline and recovery in tube fluorescence at discrete sites (Fig. 7b, black arrowheads), indicating that the underlying membrane experienced constriction and relaxation. Thus, GTP hydrolysis causes constriction which progresses to fission on a thin tube but fails at fission on a thick tube. That the constriction recovers with time could indicate scaffold disassembly. GTP addition to preassembled Drp1 ± GFP scaffolds also caused fission (Fig. 7c) and dual-channel imaging revealed that of a total of 35 single fission events analyzed, 26 events showed an intact scaffold while 9 showed a split scaffold (Fig. 7c, see Supplementary Movies 9 and 10). An intact scaffold is a consequence of fission at the edge of the scaffold and the bare tube while a split scaffold arises from fission of the underlying tube. Our results therefore reveal a bias toward fission occurring at the edge of the scaffold and the membrane. In contrast, similar analysis carried out with the classical dynamins had revealed fission to occur almost exclusively under the scaffold^44^.

### Drp1-catalyzed fission on organellar mimics

**Fig. 8:**
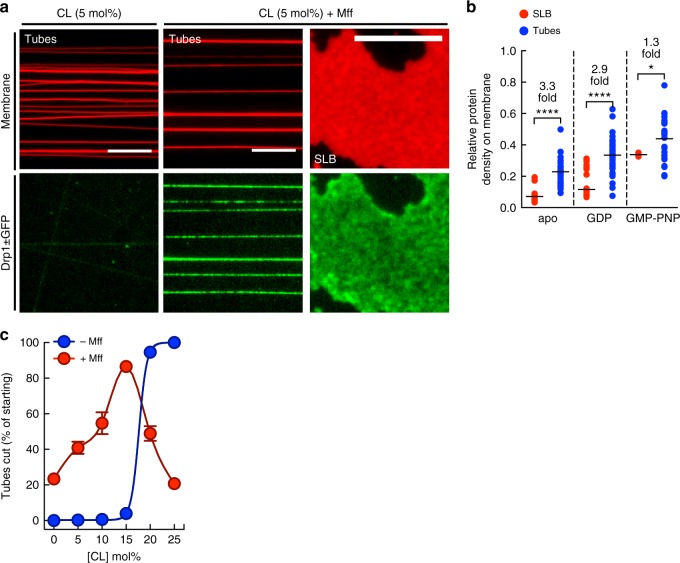
Drp1-catalyzed fission on compositional mimics of the mitochondria and peroxisomes. **a** Representative images of Drp1 ± GFP on tubes and SLB with 5 mol% CL with and without Mff. Scale bar = 10 µm. **b** Ratios of Drp1 ± GFP and membrane fluorescence on the SLB and tubes; ****p < 0.0001, *p = 0.013, Mann–Whitney’s test. *n*(SLB) = 40 and *n*(tube) = 32 from a single experiment for apo. *n*(SLB) = 43 and *n*(tube) = 46 from a single experiment for GDP. *n*(SLB) = 27 and *n*(tube) = 23 from a single experiment for GMP-PNP. **c** Plot showing the percentage of tubes cut as a function of CL concentration on tubes with and without Mff. Data represents the mean ± S.E.M. of *n*(tubes) >50 for each condition

Drp1 relies on adapter proteins on mitochondria and peroxisomes for its recruitment to the membrane^19^. To better mimic these organelles, we focused on Mff, an adapter that is critical for Drp1 recruitment^49,50^ and appears enriched at the division site^20^. We purified the cytosolic domain of Mff (Mff^ΔC20^) (Supplementary Figure 4A) and tethered it by a His tag at its C-terminus to templates containing the chelator lipid, 1,2-dioleoyl-sn-glycero-3-[(N-(5-amino-1-carboxypentyl)-iminodiacetic acid)succinyl (DGS-NTA Ni^2+^, 5 mol%)^51^. Such tethering mimics the native orientation of the tail-anchored Mff. After their formation, templates were first allowed to bind the externally added Mff and were washed with buffer to remove unbound protein. Following this, Drp1 was flowed on to the templates. On templates containing 5 mol% CL, similar to concentrations seen on the mitochondrial outer membrane^48^, the presence of Mff dramatically improved Drp1’s membrane binding both to the tubes and the SLB (Fig. 8a, b). Drp1 still showed a preference for binding the curved membranes tubes over the planar SLB (Fig. 8b) but to a lesser extent than seen earlier on templates containing CL alone (Fig. 5a). Remarkably, Drp1 with GTP also severed tubes displaying Mff and a systematic analysis of Mff-coated tubes with varying CL concentrations revealed how fission could be regulated. Thus, the minimum concentration of CL required for fission in the absence of Mff was 20 mol% (Fig. 8c, blue trace). The presence of Mff dramatically changed this requirement with ~20% of the tubes showing fission even in the absence of CL. Interestingly, Mff caused the CL dependence to now show a biphasic trend with an optimum at 15 mol% CL (Fig. 8c, red trace). This suggests that CL is not an absolute requirement for fission but depending on its concentration can either facilitate or inhibit fission. In comparison to Drp1, Dnm2 also severed Mff-coated tubes with 5 mol% CL but to a significantly lower extent (% tubes cut with Dnm2 = 25 ± 2, mean ± S.E.M, *n* = 15; % tubes cut with Drp1 = 41 ± 3, mean ± S.E.M., *n* = 20, *P* = 0.0014, unpaired t-test). We were unable to analyze fission probabilities as a function of starting tube sizes since the DGS-NTA Ni^2+^ lipid quenches *p*-Texas Red DHPE fluorescence, which interfered with the reliable estimation of starting tube sizes.

## Discussion

In this study, we first addressed whether there was a role for Dnm2 in peroxisomal fission. The results from our CRISPR/Cas9-mediated and chemical inducible knockout cell lines suggest that Dnm2 is dispensable for organelle fission, while Drp1 is essential. We have measured mitochondrial interconnectivity and fission event occurrence using live-cell imaging and concluded that the loss of Drp1, but not Dnm2, is the determining factor for hyper-connectivity and abolishment of fission events. Although Dnm2 can participate in fission events with Drp1, we do not see gross morphological changes in the mitochondrial network characteristic of global fission defects in Dnm2^KO^ cells. Loss of all dynamins 1,2,3 did not lead to elongated peroxisomes or mitochondria, suggesting a peripheral role of the classical dynamins in organelle fission. At present, we cannot explain the differences between our results and the previously published^21^. One reason could lie in the differences in cell lines and experimental approaches involving gene disruption here and siRNA knockdown used previously. We do note the apparent increased density of the mitochondrial network observed in Dnm2^KO^ cells, which might be due to reduced fission events and/or a pleiotropic cellular response caused by the loss of the core protein involved in clathrin-mediated endocytosis.

Explaining the results above, we find that Drp1 alone can execute fission on compositional mimics of mitochondrial and peroxisomal membranes in vitro. We believe our results are distinguished from earlier reports of a lack of fission by Drp1 due to the assay system of preformed membrane tubules that better mimic the mitochondrial architecture and by the careful choice of fluorescent constructs that show robust activation of GTP hydrolysis upon self-assembly. In addition, or alternatively, these could reflect intrinsic differences between Drp1 isoform 3 used here from other isoforms used earlier^13,22,23,25,26^, as the various isoforms have been previously reported to exhibit significant differences in assembly-stimulated GTPase activities^52^. Drp1 catalyzes fission by constricting the underlying tube. Indeed, recent studies point to a GTPase-dependent curling of Drp1 filaments into rings with an inner diameter of 16 nm which, taking into account the 5 nm bilayer thickness, should constrict the underlying tube to a lumen size of 6 nm^46^. Such extreme constriction is close to the limit where the bilayer would spontaneously form a hemi-fusion intermediate leading to fission^53^. Importantly, our results reveal Drp1 to be a robust fission catalyst with an intrinsic capacity to sever tubes of sizes far larger than can be severed by Dnm2. Such differences between the mitochondrial and endocytic dynamics could have evolved to accommodate the widely different substrates these GTPases act upon to catalyze fission, as has been suggested earlier^24^. Furthermore, these differences preclude the possibility that Drp1 merely functions to remodel and constrict mitochondria and thereby render Dnm2 to manage the final severing event. Given the involvement of Dnm2 in mitochondrial fission events, we suggest that Dnm2 may facilitate the process. For peroxisomes, which have a smaller starting membrane diameter, Drp1 may function alone. An overarching question, more so in the case of Drp1-catalyzed fission of wide tubes, is to elucidate if constriction of these tubes necessitates a garroting action whereby individual rungs slide past each other^54^, or a quasi-stable scaffold where the individual subunits assemble upon GTP-binding and disassemble upon GTP hydrolysis^55^, or an altogether different mechanism that converts helical scaffolds to rings upon GTP hydrolysis^46^.

Our studies show that membrane topology as well as the coincident display of protein- and lipid-binding partners can exert a significant influence on the membrane recruitment of Drp1, but once recruited Drp1 can function as a minimal apparatus requiring only membrane engagement, self-assembly and stimulated GTP hydrolysis to execute fission. Since Drp1 binding partners are constitutively present on mitochondria and peroxisomes, our results from cellular and reconstitution-based assays indicate that Drp1 is both the necessary and sufficient dynamin member for mitochondrial and peroxisomal division. The Drp1 division apparatus appears to be intrinsically regulated by membrane topology, operational on a tube thinner than 0.5 μm. This attribute would necessitate an upstream constriction process, thus explaining the role of ER-organelle contact sites^20^, for the division of mitochondria and peroxisomes.

## Methods

### Constructs and plasmids

cDNA fragments were amplified via PCR and subsequently cloned into pBMN-Z (Addgene, 1734) in place of the LacZ insert or BamHI/SalI linearized pBABE-puro (Addgene, 1764). For Dox-inducible constructs, cDNA fragments were amplified via PCR and subsequently cloned into pLVX (Clontech, 632164), using EcoRI and BamHI restriction sites. For generation of stable-expression and rescue cell lines, retroviral and lentiviral constructs were used to transduce the corresponding main clone. Point mutations were generated using the QuikChange II XL Site-Directed Mutagenesis Kit (Agilent Technologies, 200521), following the manufactures instructions. A detailed list of expression vectors used for this study can be found in Supplementary Table 2. A detailed list of all primers used in this study can be found in Supplementary Table 4. The human Drp1 isoform 3 (Uniprot ID: O00429) was a gift from Richard Youle (Addgene plasmid #45160) and Drp1 isoform 3 (K38A) was a gift from Alexander van der Bliek (Addgene plasmid #45161). The human, fly (dynamin-related protein 1, isoform A, Uniprot ID: Q9VQE0) and yeast (dynamin-related protein, Uniprot ID: Q09748) orthologs as well as the point mutants of Drp1 described here were cloned with N-terminal 6xHis and C-terminal StrepII tags in a pET15B vector by PCR. mEGFP was inserted at the N- or C-termini of Drp1 to generate GFP-Drp and Drp-GFP, respectively. Mff^Δ20^ was cloned with an N-terminal StrepII and C-terminal 6xHis tags in a pET15B vector. The 6xHis tag from Drp1 was removed by PCR for experiments on templates displaying Mff recruited via DGS-NTA Ni^2+^ lipids. All clones were confirmed by DNA sequencing.

### Generation of gene-edited HeLa and MEF cell lines

HeLa and MEF knockout cell lines (ATCC) were generated using the CRISPR/Cas9 system^56^. The target site was determined through gene analysis using CHOPCHOP^57^. CRISPR/Cas9 cell lines were sorted through fluorescence (GFP for CRISPR/Cas9) flow cytometry to give single cells to ensure clonality. The Dnm2^KO^/Drp1^KO^ line was generated from the Dnm2^KO^ parental cell line. For the genomic verification of all knockout cell lines, the targeted exons were PCR-amplified from genomic DNA isolated from individual clones. PCR products were ligated into pGEM4Z expression vectors and single clones analyzed by Sanger sequencing^58^ (Supplementary Table 1). HeLa and MEF WT cells were used as controls.

### Cell culture

All HeLa and MEF lines were cultured in Dulbecco’s modified Eagle’s medium (DMEM) containing 10% (v/v) fetal bovine serum (FBS), 1% (v/v) penicillin–streptomycin, 1% (v/v) Glutamax (Gibco), and 50 μg/ml uridine and were maintained at 37 °C in an atmosphere of 5% CO_2_. Transient transfections of HeLa cells were performed using Lipofectamine LTX with Plus Reagent or Lipofectamine 2000 (Thermo Fisher Scientific, 15338100), according to manufacturer’s instructions. Stable retroviral and lentiviral cell lines were created according to the following protocol^58^. In brief, HEK293T cells were transfected with lentiviral and retroviral constructs using Lipofectamine LTX and secreted virus were harvested 3 days after infection. Virus particles were sterile filtered (0.45 µm, PDVF, Milipore) and supplied with 1 µg/µl polybrene before added to target cells. Target cells were infected for 1 day and then the media was replaced. Stable cells were selected either via puromycin selection (2 µg/ml) or FACS sorted for the fluorescence transduced tag. Cell lines were routinely tested for mycoplasma contamination using PlasmaTest (InvivoGen). Fibroblast cultures were maintained as previously described^27,59^. Activation of the Cre-translocase was initiated with 4-hydroxytamoxifen (OHT, Sigma, H-6278)^27,59^. Cells were seeded at 25K cells/6-well and treated for 2 days with 3 µM OHT, resulting in the depletion of dynamin 1,2 and 3 at day 5–6 after treatment. Experiments were performed on cells between 7 and 9 days after seeding. Untreated cells were seeded and cultured in parallel and used for as controls for experiments. Stable cell lines expressing proteins under a doxycycline-inducible promoter (pLVX) were cultured for 2 days with 100 ng/ml doxycycline in full DMEM before analysis.

### Transferrin uptake assay

Cells were seeded the day before the assay to reach 70% confluency. Cells were washed two times with 1xPBS and starved for 1 h in serum-reduced OptiMEM medium (Thermo Fisher Scientific, 31985062) at 37 °C. Cells were incubated with 25 µM Cy3-conjugated transferrin (Jackson ImmunoResearch, 009160050) for 15 min, washed with 1xPBS, fixed and stained for mitochondria and phalloidin.

### Stress-mediated mitochondrial fission

Carbonyl cyanide 4-(trifluoromethoxy)phenylhydrazone (FCCP) (Sigma) was used at a final concentration of 20 μM in full DMEM and was applied for 2 h at 37 °C to the cells, before they were fixed and stained. For live-cell imaging of FCCP induced fission, cells were seeded two days prior to the assay in FluoroDishes (World Precision Instruments) and treated for 45 min with 20 µM FCCP before imaging.

### Antibodies

Details about antibodies used in this study can be found in Supplementary Table 3.

### Western blotting

Whole cell lysates were prepared using 1xLDS buffer (Thermo Fisher Scientific, NP0008) + 100 mM DTT and separation of proteins by Tris-Tricine SDS-PAGE was performed as previously described^60^. Signals were detected on PVDF membrane using ECL chemiluminescent substrate (Bio-Rad Laboratories) on a BioRadXRS + ChemiDoc system with Image Lab software (Bio-Rad Laboratories). See Supplementary Figure 6 for uncropped scans of blots.

### Immunofluorescence assays

Cells were fixed with 4% (w/v) paraformaldehyde in PBS (pH 7.4), permeabilised with 0.5% (w/v) Triton X-100 in PBS and incubated with primary antibodies for 60 min at room temperature. Primary antibodies were labeled with either Alexa-Fluor-488, Alexa-Fluor-568- or Alexa-Fluor-647-conjugated anti-mouse-IgG, anti-rabbit-IgG or anti-chicken-IgG secondary antibodies (Molecular Probes). F-actin was stained using a phalloidin–Alexa568 conjugate (Thermo-Fisher). Hoechst 33258 (1 μg/ml) was used to stain nuclei.

### Expression and purification of proteins

Proteins were expressed in BL21(DE3) in autoinduction medium (Formedium, UK) at 18 °C for 36 h. Cells were pelleted and stored at −40 °C. For purification, frozen bacterial pellets were resuspended in 20 mM HEPES pH 7.4 buffer with 500 mM NaCl and supplemented with protease inhibitor cocktail (Roche). Cells were lysed by sonication in an ice water bath. The lysate was spun down at 18,500 × *g* for 30 min and the supernatant was incubated with His-Pur resin (Thermo Scientific) for 1 h at 4 °C. The resin was then poured into a PD-10 column, washed with 100 ml of 20 mM HEPES pH 7.4 buffer with 150 mM NaCl and bound protein was eluted with 20 mM HEPES pH 7.4 buffer with 150 mM NaCl and 250 mM imidazole. The elution was then applied to 5 ml HiTrap Streptactin column (GE Lifesciences). To reduce nonspecific binding, the column was first washed with 20 mM HEPES pH 7.4 buffer with 300 mM NaCl followed by a wash with 20 mM HEPES pH 7.4 buffer with 150 mM NaCl. Bound protein was eluted with 20 mM HEPES pH 7.4 buffer with 150 mM NaCl, 1 mM DTT, and 2.5 mM desthiobiotin (Sigma).

### Labeling of Drp1 with extrinsic fluorophores

Drp1 was dialyzed overnight at 4 °C against 20 mM HEPES pH 7.4 buffer with 150 mM NaCl, spun down at 100,000 × *g* to remove aggregates and labeled with 10-fold molar excess of the thiol-reactive Alexa 488 C5 maleimide dye (Invitrogen, Life Technologies) for 1 h at room temperature. Reactions were quenched with 1 mM DTT. Excess free dye was removed by dialysis. The labeled protein was resolved on a 10% SDS gel and then judged to be free of unreacted dye, which typically migrates at the dye front. The typical degree of labeling achieved under these conditions was 1:1 of protein:dye (mol/mol).

### GTPase assays

Drp1 and the fluorescent constructs were dialyzed overnight at 4 °C against 20 mM HEPES pH 7.4 buffer with 150 mM NaCl and spun down at 100,000 × *g* to remove aggregates. Cardiolipin (CL) and 1,2-dioleoyl-sn-glycero-3-phosphocholine (DOPC) (both from Avanti Polar Lipids) were aliquoted in a 25:75 molar ratio into a clean glass test tube, dried under vacuum for 30 min and hydrated in 20 mM HEPES pH 7.4 buffer with 150 mM KCl for 1 h at 50 °C. Samples were vortexed vigorously to prepare multilamellar vesicles. Unilamellar vesicles were prepared by extruding the multilamellar vesicles through a 100-nm pore size filter (Avanti Polar Lipids). Drp1 (1 μM) was mixed with GTP (Jena Biosciences, Germany) (1 mM) in the absence or presence of liposomes (100 μM) in 20 mM HEPES pH 7.4 buffer with 150 mM KCl and 1 mM MgCl_2_ and incubated at 37 °C. Aliquots were taken at regular intervals and quenched with 5 mM EDTA. Inorganic phosphate released was assayed with the malachite green reagent according to ref. ^15^.

### Membrane templates

The membrane curvature- and protein binding-insensitive lipid probe *p*-Texas red DHPE was separated from the mixed isomers of Texas Red DHPE (Invitrogen) using thin-layer chromatography on silica gel plates (Sigma), as described earlier^39^. CL, DOPC, and *p*-Texas Red DHPE were reconstituted in a 25:74:1 molar ratio in chloroform and brought to a final concentration of 1 mM total lipid. For experiments to display the cytosolic domain of His-tagged Mff, lipid stocks contained DGS-NTA Ni^2+^ at 5 mol%. Lipid stocks were stored at −40 °C and brought to room temperature before use. Membrane templates were prepared as mentioned earlier^39^. Briefly, 2 μl of the lipid stock was spread on glass coverslips previously passivated by the covalent attachment of PEG8000. The coverslip was left in vacuum to dry and assembled in a flow cell (FCS2, Bioptechs, PA). Lipids were hydrated by passing 20 mM HEPES pH 7.4 buffer with 150 mM KCl. Passing the same buffer at high flow rates resulted in the formation of a supported lipid bilayer at the source where the lipid was spotted and membrane tubes downstream of the source. Buffer flow was then stopped and the tubes were allowed to settle down, which with time adhered to defects on the glass surface that resisted PEGylation. Subsequently, reactions mixtures are flowed on to these templates at 10-fold slower flow rates than was used to prepare the template. As described earlier^39^, the SLB formed during template preparation was used as an in situ calibration standard to estimate tube dimensions. This is based on the premise that fluorescence of diffraction-limited membrane tubes labeled with a fluorescent lipid analog is proportional to the net membrane area (see Supplementary Figure 5 for a step-wise description of methodology). For checking CL distribution, templates were prepared from lipid stocks containing 1 mol% of a membrane curvature-insensitive lipid BiotinCap-DOPE (Avanti Polar Lipids)^40^. CL was detected by flowing in 0.4 mM NAO (Thermo Scientific)^62^. BiotinCap-DOPE was detected using Alexa647 streptavidin. All experiments with Drp1 on membrane templates were carried out with a total protein concentration of 1 μM in the absence or presence of 1 mM nucleotides (Jena Biosciences) in 20 mM HEPES pH 7.4 buffer with 150 mM KCl and 1 mM MgCl_2_. Experiments with the human and fly Drp1 were carried out at 25 °C while those with the yeast Drp1 required 37 °C for robust fission activity. Buffers were supplemented with an oxygen scavenger cocktail when necessary^39^. For experiments with Mff, templates were prepared with DGS-NTA Ni^2+^, incubated with 1 μM protein for 10 min following which excess protein was washed off before adding Drp1.

### Microscopy

Confocal microscopy was performed on either a Leica TCS SP5 5 channel (405, 488, 543, 594, 647 nm) confocal microscope or a Leica TCS SP8 confocal microscope (405, 488, 552, 647 nm) equipped with HyD detectors using a 63× oil immersion objective. All images were processed using Fiji/ImageJ^61^. Images in all experimental groups were obtained using the same settings, except for detector gain adjustments on the Leica TCS SP5 microscope and accumulation time on the Leica TCS SP8 microscope. When required, z-sectioning was performed using 300–500-nm slices. Leica.lif files were converted to multi-color.tiff composite stacks using custom-written Fiji/ImageJ macros. Two color live-cell imaging of pBMN-BFP-Sec61 and mtDendra2 constructs was performed at 37 °C on a Leica TCS SP8 confocal laser scanning microscope (405, 488, 552, 647 nm) equipped with HyD detectors using a 63× oil immersion objective. Cells were seeded two days prior to the experiment in FluoroDishes and media was changed 1 h before the imaging. Movies of 3 µm z-stacks were acquired sequentially at 1024 × 1024 px for 7 min. Live-cell imaging of pBABE-GFP-Dnm2, pBABE-mCherry-Dnm2, and pBABE-GFP-Dnm2-K44A constructs was performed at 37 °C on a Leica TCS SP8 confocal laser scanning microscope (405, 488, 543, 647 nm) equipped with HyD detectors using a 63× oil immersion objective. Cells were seeded two days prior to the experiment in FluoroDishes. Cells were incubated for 30 min with 50 nM MitoTracker Red (Thermo Fisher Scientific, M7512), washed twice with 1xPBS, replenished with fresh media and used for the experiment. Movies of 3 µm z-stacks were acquired sequentially at 1024 × 1024 px for 7 min. Live-cell confocal microscopy of HeLa rescue cell lines stably expressing pBMN-Dendra2-SKL with pBabe-mCherry-Dnm2 and/or pLVX-mScarlet-Drp1 was performed at 37 °C on a Leica TCS SP8 confocal laser scanning microscope (405, 488, 552, 647 nm) equipped with HyD detectors using a 63× oil immersion objective. Cells were seeded two days prior to the experiment in FluoroDishes and media was changed 1 h before the imaging. Movies of 2 µm z-stacks were acquired sequentially at 1024 × 1024 px for 7 min. Live-cell two color microscope of Drp1 and Dnm2 at mitochondrial fission sites was performed at 37 °C on a Leica TCS SP8 confocal laser scanning microscope (405, 488, 543, 647 nm) equipped with HyD detectors using a 63× oil immersion objective. HeLa Dnm2^KO^ cells rescued with mCherry-Dnm2 were seeded 3 days prior to the experiment in FluoroDishes. Two days before the experiment, cells were transfected with pGFP-Drp1 using Lipofectamine LTX reagent, following the manufacturer’s instructions (Thermo Fisher Scientific, 15338100). Cells were incubated for 30 min with 50 nM MitoTracker Deep Red FM (Thermo Fisher Scientific, M22426), washed twice with 1xPBS, replenished with fresh media and used for the experiment. Movies of 2 µm z-stacks were acquired sequentially at 1024 × 1024 px for 9 min. Confocal live-cell FRAP microscopy was performed at 37 °C on an Olympus FV1000 equipped with 405, 488, 543, and 647 nm solid state pumped lasers, and a 63× water immersion objective. Cells were seeded two days prior to the experiment in FluoroDishes and media was changed 1 h before the imaging. For Dendra2 FRAP assays, time-series of multiple cells were taken in 1024 × 1024 px format, 4% 488 nm laser power, 25% 543 nm laser power and 2 µs pixel dwelling time. A circular ROI with 100 px diameter was illuminated for 2 s with the 405 nm laser at 5% power. Fluorescence recovery (=influx) of green, non-switched Dendra2 flowing back into the ROI was recoded for 7.5 min with a 15 s frame-rate and analyzed in ImageJ/Fiji. For time-lapse recording for fission events counting, 7.5-min time-series were taken in 640 × 640 px or 1024 × 1024 px format, 4% 488 nm laser power, 2 µs pixel dwelling time, 4× line averaging and 2 frames per minute. Olympus.oif files were converted to multi-color.tiff composite stacks using custom-written Fiji/ImageJ macros. Membrane templates were imaged using an Olympus IX71 inverted microscope equipped with a 100×, 1.4 NA oil immersion objective and a stable LED light source (Thor Labs). Fluorescence emission was collected through appropriate filters (Semrock) on an Evolve 512 EMCCD camera (Photometrics). Image acquisition was controlled by Metamorph software (Molecular Devices).

### Image and statistical analysis

Images were analyzed using Fiji and custom-written macros^61^. Changes in Dendra2 fluorescence upon photoconversion was enumerated using the RatioPlus plugin (https://imagej.nih.gov/ij/plugins/ratio-plus.html). Mitochondrial fission events were enumerated as follow: 7.5 min confocal time-lapses were bleach corrected, median filtered and converted into binary 8-bit files. Mitochondrial objects were then counted for each frame over time and used as basis for fission count plotting and statistical analysis. For morphological assessment of mitochondria and peroxisomes, two analytical approaches were used. First, confocal z-stacks of immunofluorescently labeled cell preparations were segmented and analyzed using the 3D ImageJ suite (http://imagejdocu.tudor.lu/doku.php?id=plugin:stacks:3d_ij_suite:start) and the 3D ROI manager. Organelle objects were de-noised, median filtered (radius 2 px) and segmented based on their intensity using Huang thresholding. Segmented images were loaded into the 3D ROI manager and segmented objects measured in 3D. Objects with a volume smaller than 2.75 px^3^, and hence considered debris and/or background, were excluded from the analysis. Second, mitochondrial and peroxisomal networks and numbers were analyzed using the MINA plugin^63^. In brief, confocal images of both mitochondrial and peroxisomal stains were skeletonized based on Otsu iterative thresholding and the resulting skeleton networks analyzed for their size, numbers and branch lengths. All graphical representations and statistical analyses were carried out on Graphpad Prism using one-way ANOVA or Student’s t-tests. For representational figures, images were median filtered (1 px) using ImageJ/Fiji. Where stated, *n*(tubes, SLB) refers to the number of tubes or SLBs analyzed.

## Electronic supplementary material

Supplementary Information

Description of Additional Supplementary Files

Supplementary Movie 1

Supplementary Movie 2

Supplementary Movie 3

Supplementary Movie 4

Supplementary Movie 5

Supplementary Movie 6

Supplementary Movie 7

Supplementary Movie 8

Supplementary Movie 9

Supplementary Movie 10

## Data Availability

The data and code that support the findings of this study are available from the corresponding authors upon reasonable request.
