# Supplementary material for: Dynamin-related protein 1 has membrane constricting and severing abilities sufficient for mitochondrial and peroxisomal fission
Source: Nat Commun. 2018 Dec 7;9:5239. doi: 10.1038/s41467-018-07543-w (PMC6286342; doi:10.1038/s41467-018-07543-w)
Supplement: Supplementary file 1 — Supplementary Information [file 41467_2018_7543_MOESM1_ESM.docx]

**Supplementary Information**

**Dynamin-related protein 1 has membrane constricting and severing abilities sufficient for mitochondrial and peroxisomal fission**

Kamerkar and Kraus et al.

**Supplementary Figure** **1.** (A) Representative confocal images of HeLa WT, Drp1^KO^, Dnm2^KO^ and Dnm2^KO^/Drp1^KO^ cells after transferrin uptake assay with Cy3-conjugated transferrin (Tfn). n(WT) = 340 cells; n(Dnm2^KO^) = 475 cells; n(Drp1^KO^) = 181 cells; n(Dnm2^KO^/Drp1^KO^) = 259 cells. Scale bar = 10 µm. Data obtained from three independent experiments. Data represents the mean ± S.E.M.; ****p<0.0001. One-way ANOVA with multiple comparisons. (B) Computational quantification of peroxisomal number in cell lines based on immunofluorescence in HeLa WT, Drp1^KO^, Dnm2^KO^ and Dnm2^KO^/Drp1^KO^ cells. n(WT) = 23 cells; n(Dnm2^KO^) = 28 cells; n(Drp1^KO^) = 28 cells; n(Dnm2^KO^/Drp1^KO^) = 27 cells. Data obtained from two independent experiments. Data represents the mean ± S.E.M.; n.s., not significant; ***p<0.001, ****p<=0.0001. One-way ANOVA with multiple comparisons. (C) Computational quantification of mitochondrial network size based on immunofluorescence in HeLa WT, Drp1^KO^, Dnm2^KO^ and Dnm2^KO^/Drp1^KO^ cells. n(WT) = 20 cells, n(Dnm2^KO^) = 20 cells, n(Drp1^KO^) = 32 cells, n(Dnm2^KO^/Drp1^KO^) = 21 cells. Data obtained from two independent experiments. Data represents the mean ± S.E.M.; n.s., not significant; ****p<0.0001. One-way ANOVA with multiple comparisons. (D) Representative confocal images of HeLa cells stably expressing GFP-Dnm2^K44A^ after transferrin uptake assay and immunofluorescent labeling for HSP60 (mitochondria) and GFP (GFP-Dnm2^K44A^). Tfn and GFP intensities of selected cells are plotted on the right. Scale bar = 10 µm. (E) Statistical quantification of Drp1 levels in HeLa WT and Dnm2^KO^ cell lines based on western blot analysis of whole cell lysates. Actin was used as a loading control and normalization. n(WT) = 9, n(Drp1^KO^) = 9. Data obtained from three independent experiments. Data represents the mean ± S.E.M.; n.s., not significant. Mann-Whitney test. (F) Representative confocal images of HeLa cells stably expressing GFP-Dnm2^K44A^ after immunofluorescence labeling for HSP60 (mitochondria) in conjunction with GFP (GFP-Dnm2^K44A^); and catalase (peroxisomes) in conjunction with GFP (GFP-Dnm2^K44A^). Scale bar = 10 µm. (G) Confocal images of immunofluorescently labeled peroxisomes (Pex14) in HEK293T WT and GFP-Dnm2^K44A^ expressing cells. Scale bar = 10 µm.

**
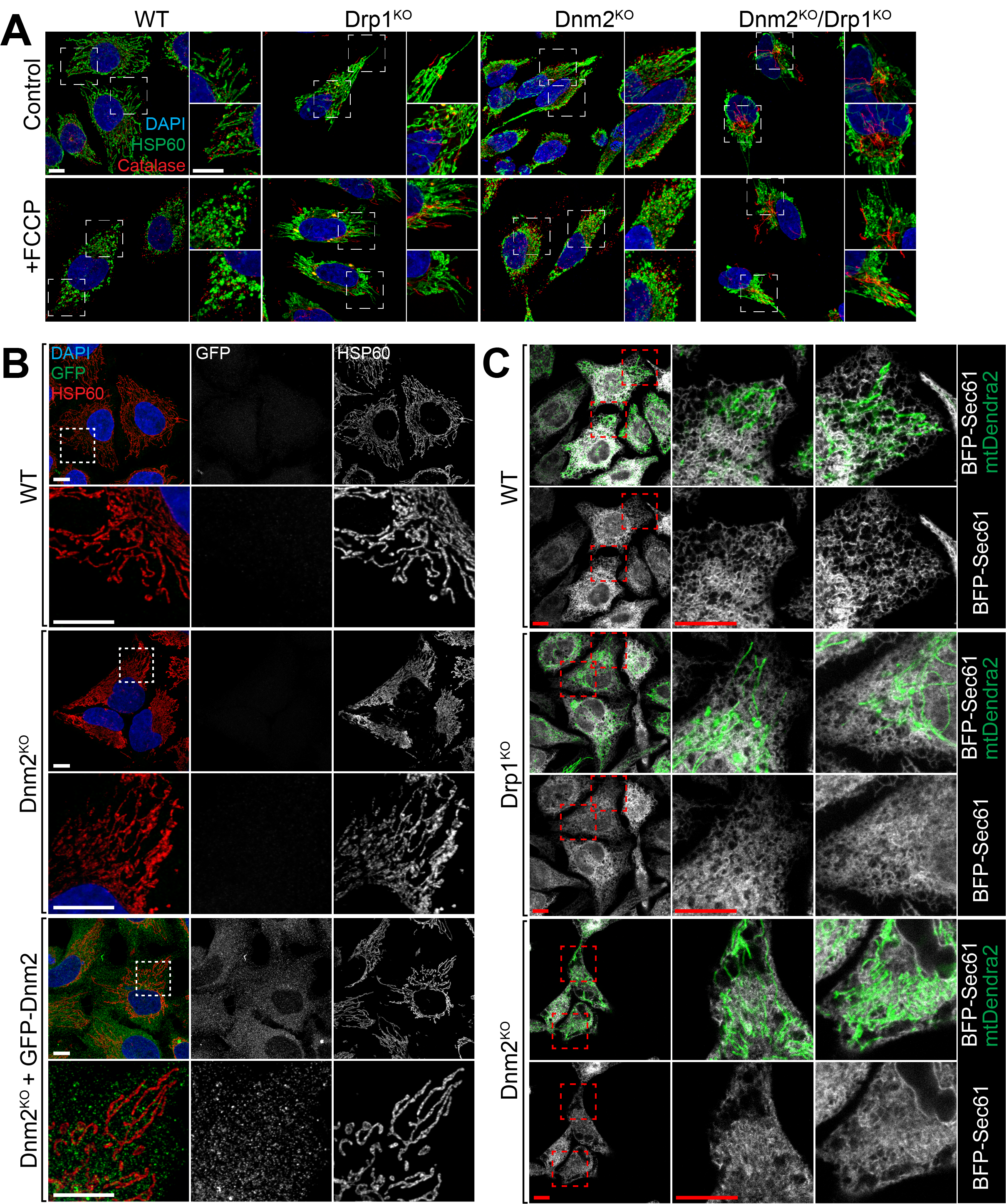
**

**Supplementary Figure 2.** (A) Confocal images of FCCP treated (20 µM) and immunofluorescently stained HeLa WT, Drp1^KO^, Dnm2^KO^ and Dnm2^KO^/Drp1^KO^ cells for mitochondria (HSP60) and peroxisomes (catalase). Scale bar = 10 µm. (B) Confocal images of HeLa WT, Dnm2^KO^ and Dnm2^KO^-GFP-Dnm2 rescue cells immunofluorescently labeled for mitochondria (HSP60) and GFP (anti-GFP). An enlargement of the hatched box is shown on the right of each panel. Scale bar = 10 µm. (C) Confocal images of HeLa WT, Drp1^KO^ and Dnm2^KO^ cells stably expressing BFP-Sec61 and mtDendra2. An enlargement of the hatched box is shown on the right of each panel. Scale bar = 10 µm.

**
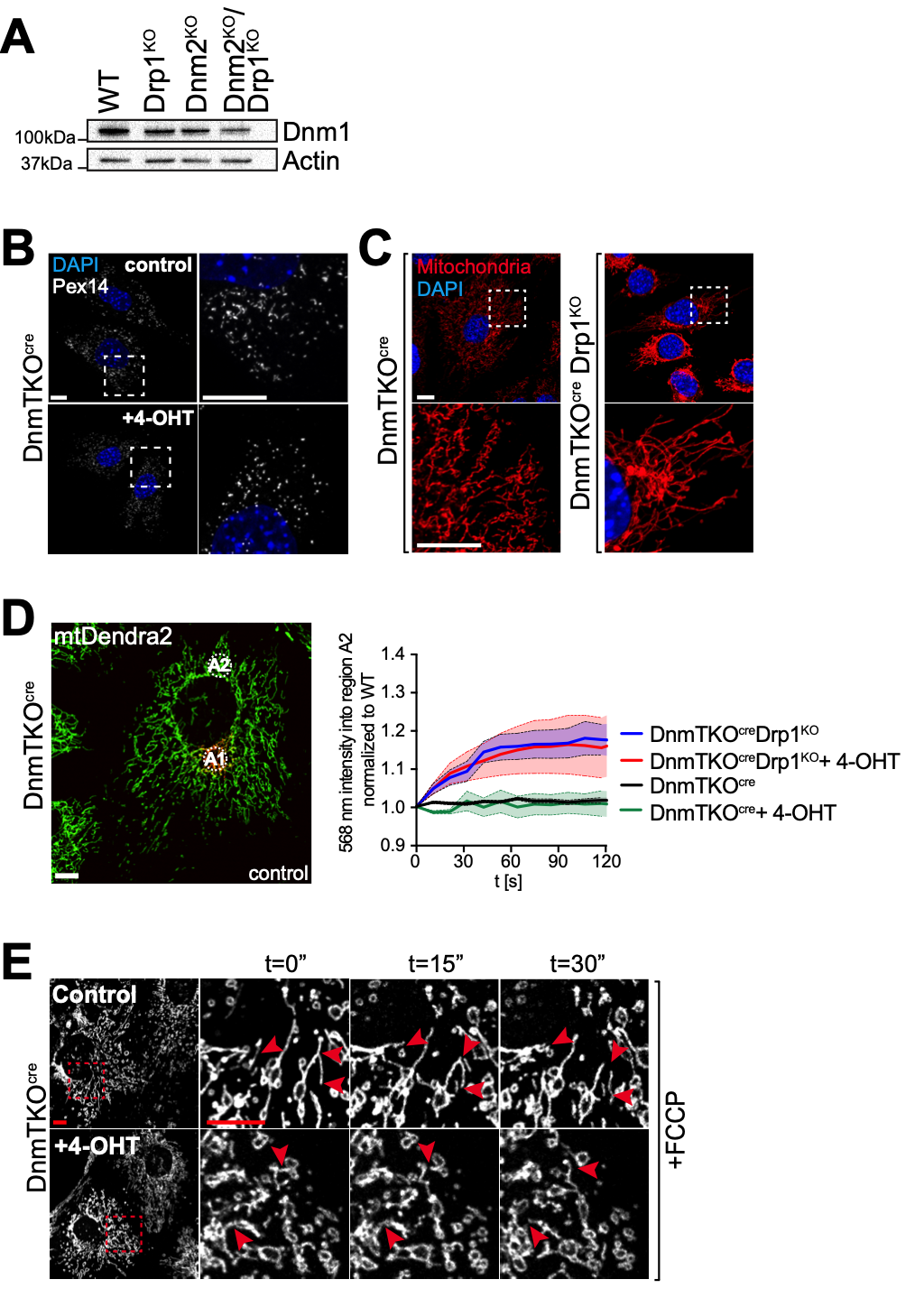
Supplementary Figure 3.** (A) Western blot analysis for Dynamin-1 in HeLa WT, Drp1^KO^, Dnm2^KO^ and Dnm2^KO^/Drp1^KO^ whole cell lysates. Actin was used as a loading control. (B) Confocal images of MEF DnmTKO^cre^ control and 4-OHT induced cells subjected to immunofluorescence and stained for DAPI (DAPI; nucleus) and peroxisomes (Pex14). An enlargement of the hatched box is shown on the right of each panel. Scale bar = 10 µm. (C) Confocal images of MEF DnmTKO^cre^ and DnmTKO^cre^+Drp1^KO^ cells subjected to immunofluorescence and stained for DAPI (DAPI; nucleus) and mitochondria (NDUFAF2). An enlargement of the hatched box is shown below each panel. Scale bar = 10 µm.

(D) Diffusion kinetics of mtDendra2 over the mitochondrial network in MEF DnmTKO^cre^, DnmTKO^cre^+Drp1^KO^ control and 4-OHT induced cells. A1 indicates the photo-conversion ROI. A2 indicates the inflow ROI at the opposite site of the photoconversion where influx of switched Dendra2 is measured. n(DnmTKO^cre^) = 23 cells; n(DnmTKO^cre^+4OHT) = 31 cells; n(DnmTKO^cre^+Drp1^KO^) = 26 cells; n(DnmTKO^cre^+Drp1^KO^+4OHT) = 23 cells. (E) Live-cell image stills of FCCP treated (20µM) MEF DnmTKO^cre^ control and 4-OHT-induced KO cells stably expressing mtDendra2. Inserts depict mitochondrial fission events during the time-lapse (red arrowheads). Scale bar = 10 µm.

**Supplementary Figure 4.** (A) Coomassie Brilliant Blue-stained SDS-PAGE of purified proteins used in this study. (B) GTPase activities of WT and various fluorescent constructs of Drp1 either alone or with liposomes of the indicated composition. Data represents the mean ± S.D. of N = 3 experiments for each construct. (C) Distribution of fluorescent Drp1 constructs on membrane tubes.

**Supplementary Figure 5.** Stepwise description of the methodology used to calculate tube sizes.

**Supplementary Figure** **6.** (A) Whole, uncropped blots incubated with antibodies against Dnm1, Dnm2, Drp1 and Actin merged with an upper white image for Figure 1a and Supplementary Figure 3a. (B) Whole, uncropped blots incubated with antibodies against Dnm2, Drp1 and Actin merged with an upper white image for Figure 3a.

**Supplementary Table 1.** Knockout cell lines created for this study.


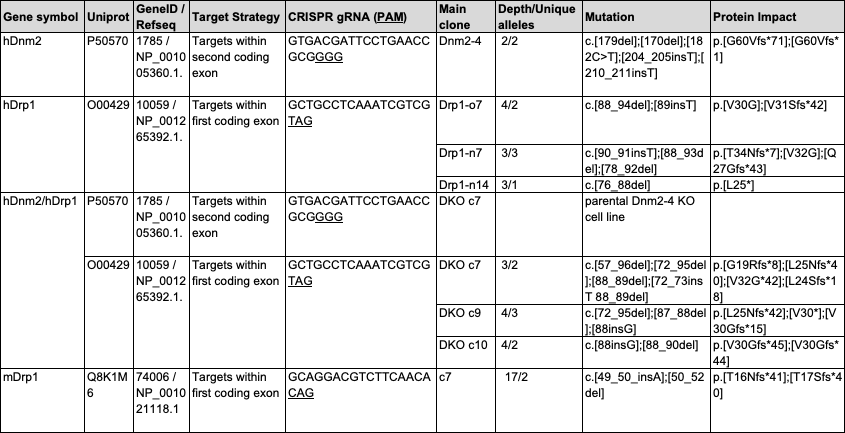


**Supplementary Table 2.** Mammalian transient, lentiviral and retroviral expression vectors and details.

**Supplementary Table 3.** Information regarding antibodies used in this study for immunofluorescence and western blotting**.**

**Supplementary Table 4.** Information about primers used in the study.
