# Supplementary material for: Dynamin-related protein 1 has membrane constricting and severing abilities sufficient for mitochondrial and peroxisomal fission
Source: Nat Commun. 2018 Dec 7;9:5239. doi: 10.1038/s41467-018-07543-w (PMC6286342; doi:10.1038/s41467-018-07543-w)
Supplement: Supplementary file 2 — Description of Additional Supplementary Files [file 41467_2018_7543_MOESM2_ESM.docx]

Description of Additional Supplementary Files.

Supplementary Movie 1. Representative time-lapse movie of Drp1 mediated peroxisomal fission in HeLa Dnm2^KO^/ Drp1^KO^ cells expressing mScarlet-Drp1.

Supplementary Movie 2. Representative 7 min time-lapse movie of mitochondrial targeted Dendra2 photoconversion experiments in HeLa WT cells.

Supplementary Movie 3. Representative 7 min time-lapse movie of mitochondrial targeted Dendra2 photoconversion experiments in HeLa Drp1^KO^ cells

Supplementary Movie 4. Representative 7 min time-lapse movie of mitochondrial targeted Dendra2 photoconversion experiments in HeLa Dnm2^KO^ cells.

Supplementary Movie 5. Representative 7 min time-lapse movie of mitochondrial targeted Dendra2 photoconversion experiments in HeLa Dnm2^KO^/Drp1^KO^ cells.

Supplementary Movie 6. Representative time-lapse movie showing membrane tubes undergoing fission with Drp1 and GTP.

Supplementary Movie 7. Representative time-lapse movie showing a tube in solution undergoing fission with Drp1 and GTP.

Supplementary Movie 8. Representative time-lapse movie showing the effect of GTP addition to preassembled Drp1 scaffolds.

Supplementary Movie 9. Representative dual-channel time-lapse movie showing fission at the edge of a preassembled Drp1±GFP scaffold. Top panel shows Drp1±GFP and bottom panel shows tube fluorescence. Time interval between frames is 0.1 s.

Supplementary Movie 10. Representative dual-channel time-lapse movie showing fission under the Drp1±GFP scaffold. Top panel shows Drp1±GFP and bottom panel shows tube fluorescence. Time interval between frames is 0.1 s.
